# Third-Degree Atrioventricular Block as the Initial Presentation of Lyme Disease

**DOI:** 10.7759/cureus.9574

**Published:** 2020-08-05

**Authors:** Sana Riaz, Alisha Garel, Abinash Subedi, Emad Mogadam, Andrew Weinberg

**Affiliations:** 1 Internal Medicine, State University of New York Upstate Medical University, Syracuse, USA; 2 Cardiology, University of Southern California, Los Angeles, USA; 3 Cardiology, State University of New York Upstate Medical University, Syracuse, USA

**Keywords:** lyme disease, lyme carditis, third-degree av block, erythema migrans

## Abstract

Lyme disease is a multisystemic infection that can present as localized disease, early disseminated, or late disease. During the early disseminated phase of Lyme disease, the hematogenous spread can result in extracutaneous manifestations, including cardiac, neurological, and joint. Lyme carditis is an uncommon manifestation occurring in patients with untreated Lyme disease. Third-degree atrioventricular (AV) block is a rarer entity. We present a case of a 21-year-old female with no significant past medical history admitted with third-degree AV block and thereby highlighting this uncommon presentation.

## Introduction

Lyme disease (LD) is caused by a Gram-negative spirochete bacteria acquired from rodent host reservoirs and transmitted by the *Ixodes* tick. The predominant bacteria responsible for LD in North America is *Borrelia burgdorferi*. The other species known to cause LD include *B. garinii, B. afzelii, and B. spielmanii* [1]. The incidence of LD in the United States is approximately 300,000 per year. Most cases are concentrated in the Northeast and upper Midwest, with 14 states accounting for nearly 96% of cases reported to Centers for Disease Control and Prevention (CDC) [2]. During the early disseminated phase of LD, hematogenous spread results in cardiac and neurological manifestations [1]. Lyme carditis (LC) is an uncommon manifestation of disseminated LD, and third-degree atrioventricular (AV) block is a rarer finding. Nearly 4%-10% of untreated patients with LD develop LC, and approximately 1% of patients with LD have second- or third-degree AV conduction blockade [3]. We present a case of a healthy 27-year-old female with no prior cardiac history and no family history of structural heart disease admitted to the hospital with third-degree heart block as the initial presentation of LD. 

## Case presentation

**Figure 1:**
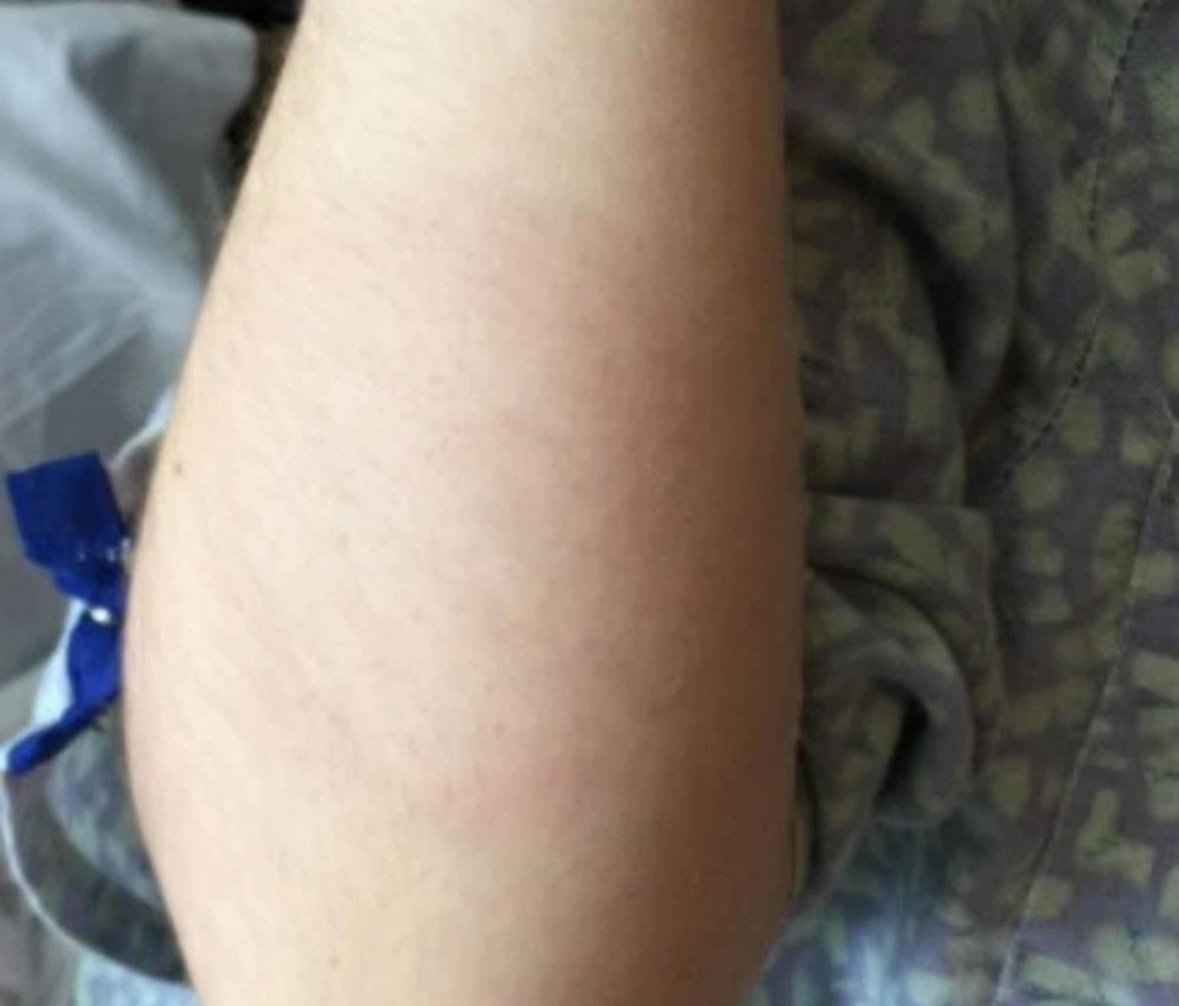
Erythema migrans in right upper extremity

A 27-year-old female from Vermont with no significant medical history presented with complaints of a one-week history of fevers, chills, migratory joint pain, and palpitations. On admission, the patient had a temperature of 38.5^⸰^C, blood pressure 109/69 mmHg, heart rate 68 bpm, and normal oxygen saturation. Physical exam was notable for erythema migrans on her back, left leg, and right arm (Figure 1). Laboratory work-up was notable for a hemoglobin of 9.1 g/dL, hematocrit 27.5%, Lyme IgM positive, thyroid stimulating hormone 0.984 U/mL, and troponin T <0.01 ng/mL. 

**Figure 2:**
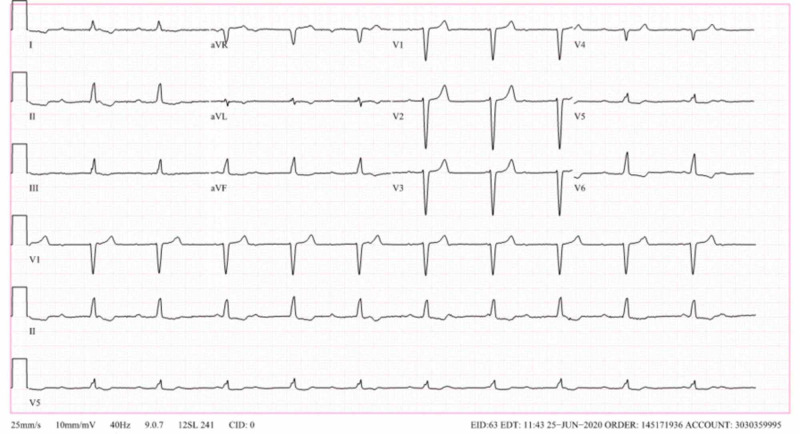
Sinus rhythm with atrioventricular dissociation and junctional rhythm

Electrocardiogram showed sinus rhythm with complete heart block (Figure 2). Transthoracic echocardiogram showed normal systolic function with an ejection fraction of 59% without wall motion or valvular abnormalities. She was treated with ceftriaxone for the management of early disseminated LD. Following the initial presentation, she was noted to be hypotensive with a systolic blood pressure of 70 mmHg, which was considered to be a Jarisch-Herxheimer reaction with the onset of treatment with ceftriaxone in the setting of acute Lyme infection. 

**Figure 3:**
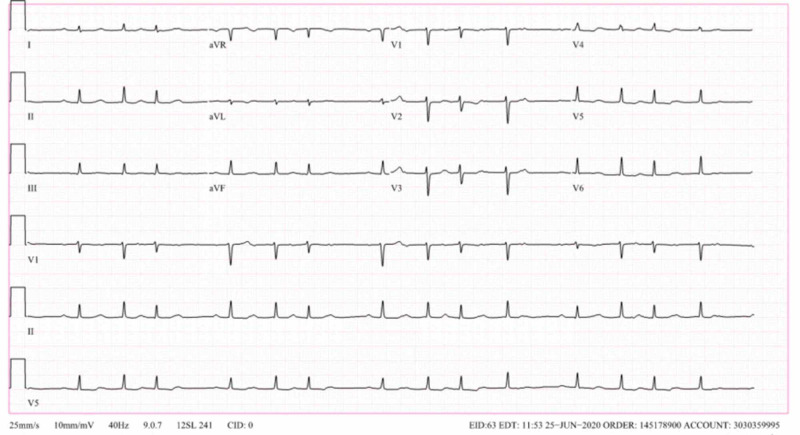
Sinus rhythm with second-degree atrioventricular block (Mobitz 1)

**Figure 4:**
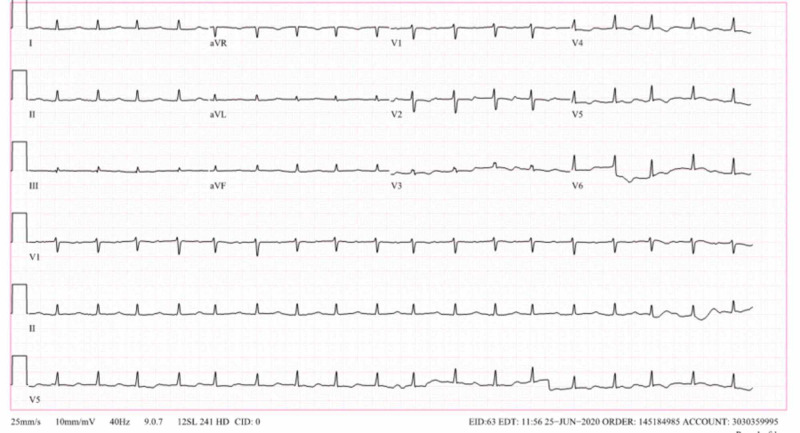
Sinus tachycardia and first-degree atrioventricular block

Day 1 post-admission, the patient’s rhythm improved initially to second-degree heart block (Figure 3), and then first degree (Figure 4) with subsequent complete resolution of heart block as well as hypotension within 48 hours post-admission. She was discharged on a four-week course of ceftriaxone. At her outpatient cardiology follow-up visit, she was asymptomatic and maintaining sinus rhythm. 

## Discussion

LC is defined as acute AV conduction disturbance, usually above the bundle of His, myocarditis, or pancarditis. The most common presentation of LC is varying degrees of AV conduction block with the most severe form being complete AV block, which occurs within one month after the onset of infection [4,5]. LC has a predilection for young males with a male to female ratio of 3:1, and this difference in gender is more pronounced among patients with third-degree AV block. Additionally, there is a bimodal distribution of LD, with the highest incidence in children aged five to nine years and adults aged 45-59 years. Patients with third-degree AV block from LC are predominantly aged 10-45 years. Another notable feature of LC is the lower frequency of associated characteristic rash. In the retrospective study by Forrester and Mead, it was noted that erythema migrans developed in only 44% of patients with third-degree heart block compared to 70%-80% in patients diagnosed with early localized LD [3]. Meanwhile, only 10%-20% of patients will develop multiple erythema migrans rashes, such as those appreciated in our case report [6]. The electrocardiographic findings in LC are variable and frequently range from prolonged PR interval to AV dissociation to complete heart block within minutes or days, as noted in our patient [7].

The pathophysiology of AV block in patients with LC is unclear but postulated to be secondary to host inflammatory response directed at the spirochetes in cardiac tissue. A lymphoplasmacytic linear perivascular and interstitial “band-like” pattern is observed on the histopathologic exam, often involving all heart layers [3]. 

The Suspicious Index in Lyme Carditis (SILC) score is a novel risk score that helps evaluate the likelihood of a patient’s high-degree AV block being secondary to LC. The score assigns weights to risk factors, namely constitutional symptoms (fever, malaise, arthralgia, and dyspnea), outdoor activity/endemic area, male gender, tick bite, age <50 years, and presence of erythema migrans. The final summed score classifies patients to be low risk (0-2), intermediate-risk (3-6), or high risk (7-12). The implementation of this risk stratification tool helps prompt recognition of LC in patients with high-degree AV block. Patients considered to be an intermediate or high risk by the SILC score require serological tests to confirm LD and intravenous antibiotics [8].

Treatment for LC helps shorten the overall duration of cardiac irregularities and prevent further complications of LD. Patients who have symptomatic second- or third-degree AV block or prolonged PR interval of ≥300 milliseconds are typically hospitalized and treated with intravenous antibiotics, preferably ceftriaxone [9]. Intravenous antibiotics are usually continued until the high-grade AV block has resolved, and the PR interval has become less than 300 milliseconds. The total length of therapy ranges from 21 to 28 days. It is imminent to establish the diagnosis of LD in young patients presenting with high-degree AV blocks so that antibiotics can be quickly and appropriately administered, and placement of a permanent pacemaker can be avoided in patients with severe conduction disturbances due to infection [9].

## Conclusions

Our case report of a young female with no prior cardiac history and no known past history of LD or tick bite presenting from an endemic area with third-degree AV block on electrocardiogram along with erythema migrans is a unique case. As mentioned above, the third-degree AV block is a rare finding and has a predilection for the male gender. Additionally, erythema migrans, along with third-degree AV block, is seen only in approximately 40% of the patients compared to 70%-80% in patients diagnosed with LD. Our case emphasizes the importance of prompt recognition of LC, thereby leading to early intervention of antibiotics and avoiding invasive intervention such as pacemaker placement. 
